# Effect of SARS-CoV-2 Infection on Selected Parameters of the Apelinergic System in Repeat Blood Donors

**DOI:** 10.3390/biomedicines12112583

**Published:** 2024-11-12

**Authors:** Marta Stanek, Anna Leśków, Dorota Diakowska

**Affiliations:** 1 Regional Center of Transfusion Medicine and Blood Bank, 50-345 Wrocław, Poland; 2 Division of Medical Biology, Faculty of Nursing and Midwifery, Wroclaw Medical University, 50-368 Wrocław, Poland; dorota.diakowska@umw.edu.pl

**Keywords:** SARS-CoV-2, virus, apelin, elabela, blood donor

## Abstract

**Background**: SARS-CoV-2 enters cells primarily by binding to the angiotensin-converting enzyme 2 (ACE2) receptor, thereby blocking its physiological functions, affecting the apelinergic system, and inhibiting the cleavage of its peptides. The appropriate concentration of peptides in the apelinergic system influences the maintenance of homeostasis and protects against cardiovascular diseases. In our research, we determined the level of selected parameters of the apelinergic system—apelin (AP), elabela (ELA), and the apelin receptor (APJ)—in repeat blood donors. **Methods**: We analyzed 120 serum samples obtained from 30 repeat donors (study group) within four time periods after a SARS-CoV-2 infection: <60 days, 61–90 days, 91–120 days, and >120 days. We compared the results from the study groups with those of the control group, which consisted of 30 serum samples collected from donors donating blood in the years 2018–2019. **Results**: We observed that the AP, ELA, and APJ concentrations in the control group are higher than in any period in the study group. In the study group, the concentrations of AP and ELA increased in subsequent study periods. AP and ELA concentrations were lower shortly after SARS-CoV-2 transfection and then slowly increased in subsequent periods. APJ concentrations, on the other hand, were lowest at 61–90 days after the infection, but the decrease, relative to their level in healthy subjects, was significant in every period studied. **Conclusions**: The results suggest that infection with SARS-CoV-2 causes changes in the parameters of the apelinergic system, both after a short period of time has passed since the onset of the SARS-CoV-2 infection, and even up to 4 months after the infection.

## 1. Introduction

Since the end of 2019, the world has been struggling with the consequences of the spread of the severe acute respiratory syndrome coronavirus 2 (SARS-CoV-2) virus and the global coronavirus disease 2019 (COVID-19) pandemic that it caused. Although today we can successfully use vaccines to prevent the rapid spread of the virus and, if infection occurs, limit the negative course of the disease [1,2], there are still many unknowns about SARS-CoV-2 virus infections and their distant consequences for the human organism.

Crucial to preventing the spread and understanding the impact of the SARS-CoV-2 virus on the human body is the discovery of the pathway of its entry into the body via the angiotensin-converting enzyme 2 (ACE2) [3,4,5,6]. The main physiological function of this integral membrane protein is the conversion of angiotensin (Ang) II into Ang 1–7 and Ang I into Ang 1–9, resulting in vasodilatation, lower blood pressure, and reduction in water and salt retention, which prevents the development of hypertension. Thus, the blocking of ACE2 by SARS-CoV-2 leads to the inhibition of Ang II conversion, contributing to inflammation and vasoconstriction, thus worsening the condition of patients with COVID-19. At the same time, ACE2 can cleave apelin (AP) peptides [7,8,9]. AP, with another ligand, elabela (ELA), and their receptor (APJ), make up the apelinergic system [10,11]. Currently, the amino acid sequence of several isoforms of APJ ligands has been discovered and determined, and apelin-13 and apelin-17, as well as ELA, were found to play a significant role in heart muscle tissue. Apelin peptides promote angiogenesis, vasodilatation, lower blood pressure, and, in general, protect against hypertension, atherosclerosis, and other chronic circulatory system disease, preventing emergencies [7,10,11,12,13]. It has been shown that a decrease in apelin levels, especially apelin-13, may promote inflammation and oxidative cell injury [14]. In COVID-19 patients (during the syndrome phase), the apelin-13 level may be increased due to ACE2 downregulation, causing the activation of angiogenesis and anti-inflammatory and antiapoptotic effects. This could prevent the occurrence of severe COVID-19 conditions and reduce its negative, long-lasting consequences (also known as long COVID or post-COVID) [5,8]. Therefore, normal or increased levels of certain apelins are necessary to maintain homeostasis.

Studies on the apelinergic system in patients with COVID-19 have previously been performed [15,16,17]; however, these studies are performed on sick patients (in whom the SARS-CoV-2 was detected by RT-PCR at the time of sampling for the study). Additionally, the control groups were recruited at the same time as the study groups (according to the information presented in these articles), and, despite a negative RT-PCR test at the time of recruitment, it is not certain that these people have never had COVID-19, which could have affected the level of the parameters studied. In our case, we recruited people who had already recovered (obtaining a negative test result at the time of sampling) and compared their plasma with the control group, from whom samples were taken before the pandemic. This allowed us to assume that people from the control group had never experienced any contact with SARS-CoV-2. No studies have been carried out to date on the levels of the components of the apelinergic system in healthy individuals after SARS-CoV-2 infection, and the results obtained have not been compared with samples taken from individuals who had no chance of becoming infected by SARS-CoV-2.

One of the healthiest social groups in terms of maintained body homeostasis is the group of repeat blood donors [18,19,20]. Due to the lack of available data, we sought to determine the levels of AP, ELA, and APJ in repeat blood donors by comparing samples taken from individuals who had recovered from COVID-19 and from those who had never had the disease. We also aimed to determine whether changes in the tested parameters are dependent on the time that has passed since the detection of the SARS-CoV-2 infection. The collected results will help answer the question of whether infection with SARS-CoV-2 can change the concentrations of selected parameters, thereby affecting the course of the infection and general human health.

## 2. Materials and Methods

### 2.1. Criteria for Samples Collection

Serum samples from 394 repeat blood donors were collected at the Regional Center of Blood Donation and Blood Treatment in Wroclaw, Poland (RCBDBT-W), after obtaining consent from the Bioethics Committee (approval no. KB-536/2022). Samples were collected from individuals meeting the criteria, which were based on European recommendations, including the European Commission Directorate—General for Health and Food Safety of 4 April 2020, developed by the National Consultant for Transfusiology’s team and agreed upon by the public blood service continuity management working group. We described the detailed criteria in our previous work [21], and the most important of them are included below:

- The donor had to have a confirmed (positive result of RT-PCR test) SARS-CoV-2 infection in the past;
- The donor had to be healthy in each study period, i.e., not be a carrier and have no symptoms of COVID-19 or any other disease;
- The donor had to meet the basic donation criteria regarding their age, weight, blood pressure, heart rate, and hemoglobin concentration (described in the regulation of the Minister of Health [22]).

**Figure 1:**
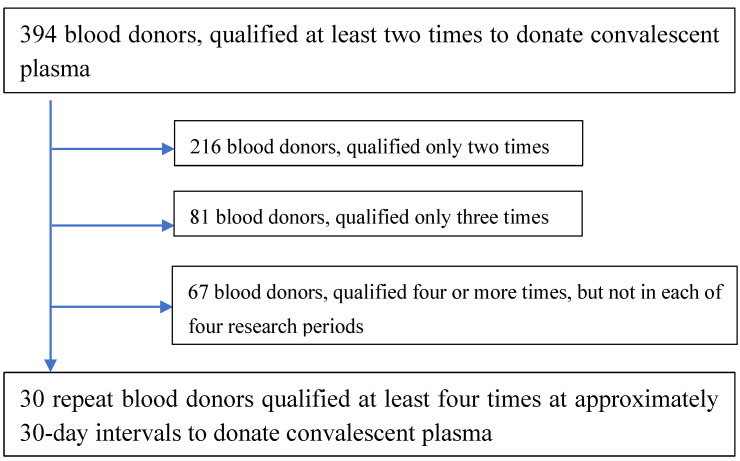
Selection of the study group.

Our research is a continuation of a project that analyzed laboratory parameters of 394 repeat blood donors [21]. Among 394 donors, 30 donated convalescent plasma at regular intervals of 30 ± 2 days. The selection process for the study group is shown in Figure 1. We selected samples that would allow us to obtain 4 samples from each donor. Based on this criterion, we selected 120 samples belonging to 30 donors, which we then analyzed. These repeat blood donors donated blood during 4 time periods after SARS-CoV-2 infection: Period 1—≤60 days; Period 2—61–90 days; Period 3—91–120 days; Period 4—>120 days.

We also used archived research material (for which we also received the commission’s consent), which consisted of 30 serum samples remaining after blood donation by individuals in the years 2018–2019 (before COVID-19).

### 2.2. Apelinergic System Parameters Determination

Blood samples were collected in BD VACUTAINER SST II Advance serum tubes with separating gel (Beckton-Dickinson, Plymouth, UK) and then centrifuged at 3000 rpm for 5 min. The obtained supernatant was transferred to 1.5 mL plastic tubes and stored at −20 °C. Control serum samples were obtained based on the same protocol. Both control and study samples were not exposed to any freeze–thaw cycles.

ELISA test kits were used to determine selected parameters of the apelinergic system, such as AP, ELA, and APJ (Shanghai Sunred Biological Technology Co., Ltd., Shanghai, China). The sensitivity of the AP assay was 0.756 ng/L, of the ELA assay was 13.703 pg/mL, and of the APJ assay was 0.288 ng/mL. The measurements were performed in triplicate in accordance with the manufacturer’s instructions.

### 2.3. Data Analysis

Categorical variables are presented as the number of events and their respective percentages. For continuous variables, the median and interquartile range (IQR) were calculated. The Mann–Whitney test was used for comparisons of two independent groups. Friedman’s ANOVA analysis was performed for comparison of data between more than two dependent samples, and the paired-samples sign test was used as a post hoc test. Correlation analyses were made using the Spearman test. Differences were considered statistically significant when *p* < 0.05. Data were analyzed using Statistica v. 13.3 (Tibco Software Inc., Palo Alto, CA, USA).

## 3. Results

Detailed characteristics of the study group are described in Table 1. Of the 30 repeat donors who participated in the study, the vast majority were men (86.7%). The level of anti-SARS-CoV-2 antibodies was determined with the use of a screening assay for detecting IgG antibodies against the antigen (protein S) of the SARS-CoV-2 virus (Euroimmun, Lubeck, Germany), describing it as low or high, based on previously published criteria [21].

**Figure 2:**
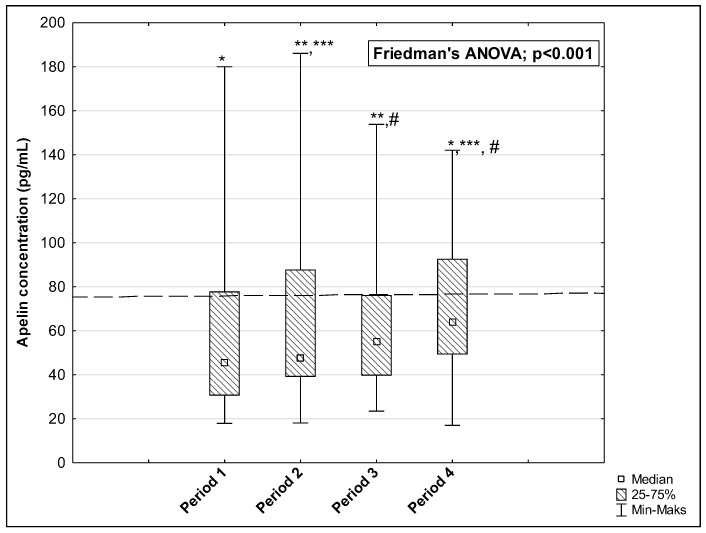
The concentration of AP in the serum of repeat blood donors in 4 time periods after SARS-CoV-2 infection. Horizontal dashed line indicates the average concentration of the tested parameter determined in the control group. *: Period 1 vs. Period 4, *p* = 0.004; **: Period 2 vs. Period 3, *p* = 0.006; ***: Period 2 vs. Period 4, *p* < 0.001; #: Period 3 vs. Period 4, *p* < 0.001.

**Figure 3:**
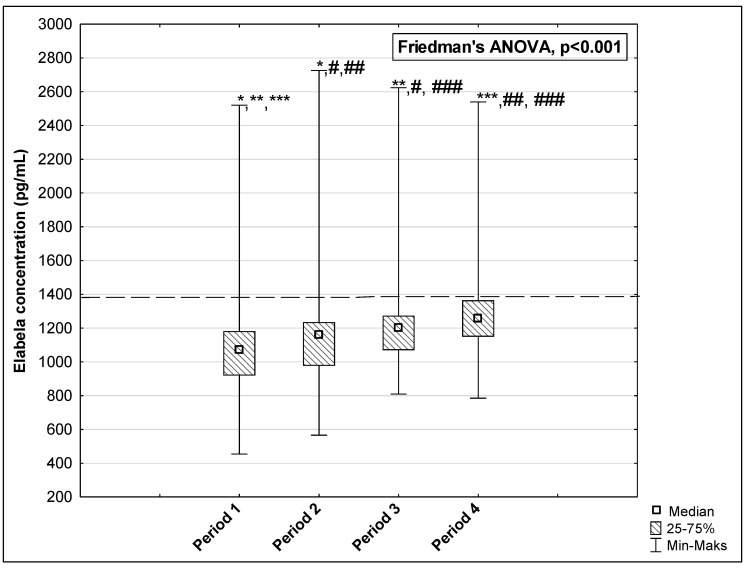
The concentration of ELA in the serum of repeat blood donors in 4 time periods after SARS-CoV-2 infection. Horizontal dashed line indicates the average concentration of the tested parameter determined in the control group. *: Period 1 vs. Period 2, *p* = 0.002; **: Period 1 vs. Period 3, *p* < 0.001; ***: Period 1 vs. Period 4, *p* < 0.001; #: Period 2 vs. Period 3, *p* = 0.017; ##: Period 2 vs. Period 4, *p* = 0.002; ###: Period 3 vs. Period 4, *p* = 0.002.

**Figure 4:**
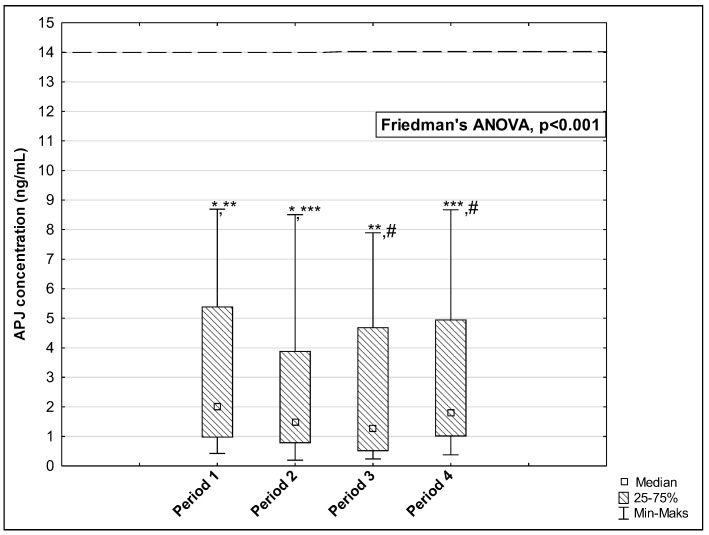
The concentration of APJ in the serum of repeat blood donors in 4 time periods after SARS-CoV-2 infection. Horizontal dashed line indicates the average concentration of the tested parameter determined in the control group. *: Period 1 vs. Period 2, *p* = 0.002; **: Period 1 vs. Period 3, *p* = 0.002; ***: Period 2 vs. Period 4, *p* = 0.044; #: Period 3 vs. Period 4, *p* = 0.002.

The results of measurements of the AP, ELA, and APJ concentrations in the studied periods are presented in Figure 2, Figure 3 and Figure 4. The concentration of AP and ELA increased depending on the length of time that passed since the onset of the disease. In the first studied period, the median AP concentration was 45.37 pg/mL (IQR: 30.60–77.91 pg/mL) and increased to 47.55 pg/mL (IQR: 39.08–87.83 pg/mL) in Period 2, to 55.18 pg/mL (IQR: 39.66–76.25 pg/mL) in Period 3, and to 63.82 pg/mL (IQR: 49.30–92.70 pg/mL) in Period 4 (*p* < 0.001) (Figure 2). Post hoc tests showed that significant differences were observed between medians of AP concentration in: Period 1 vs. Period 4, *p* = 0.004; Period 2 vs. Period 3, *p* = 0.006; Period 2 vs. Period 4, *p* < 0.001; and Period 3 vs. Period 4, *p* < 0.001. The median ELA concentration was 1071.50 pg/mL (IQR: 920.00–1182.00 pg/mL) in Period 1 and increased to 1157.50 pg/mL (IQR: 978.00–1235.00 pg/mL) in Period 2, to 1203.50 pg/mL (IQR: 1070.00–1274.00 pg/mL) in Period 3, and to 1255.50 pg/mL (IQR: 1150.00–1365.00 pg/mL) in Period 4 (*p* < 0.001, Figure 3). There were significant differences between Period 1 and Period 2, *p* = 0.002; Period 1 and Period 3, *p* < 0.001; Period 1 and Period 4, *p* < 0.001; Period 2 and Period 3, *p* = 0.017; Period 2 and Period 4, *p* = 0.002; and Period 3 and Period 4, *p* = 0.002. In the case of APJ, the highest concentration was observed in Period 1 up to 60 days after the onset of the disease (2.01 ng/mL (IQR: 0.96–5.39 ng/mL)), which then decreased in Period 2 to 1.48 ng/mL (IQR: 0.77–3.89 ng/mL) and further to 1.27 ng/mL (IQR: 0.50–4.69 ng/mL) in Period 3. A higher level was reached in Period 4 after 120 days: 1.82 ng/mL 1.82 ng/mL (IQR: 1.00–4.96 ng/mL) (*p* < 0.001, Figure 4). We observed significant differences between the following time periods: Period 1 vs. Period 2, *p* = 0.002; Period 1 vs. Period 3, *p* = 0.002; Period 2 vs. Period 4, *p* = 0.044; and Period 3 vs. Period 4, *p* = 0.002.

We also analyzed whether observed changes were significant between Period 1 and Period 4 and as related to the control group. Periods 1 and 4 were selected to determine the quintessential relationship between the parameters studied immediately after recovery from SARS-CoV-2 infection and in the most distant of the studied periods, over 120 days after infection, compared to the control. The data we obtained are presented in Table 2.

We observed that AP, ELA, and APJ concentrations in the control groups were higher than in any period in the study group. The findings were statistically significant when comparing the following: control vs. AP concentration in Period 1 (*p* = 0.005), control vs. ELA concentration in Period 1 (*p* = 0.039), control vs. APJ concentration in Period 1 (*p* < 0.001), and control vs. APJ concentration in Period 4 (*p* < 0.001). There were also significant differences in AP and ELA concentrations from Period 1 to Period 4.

**Figure 5:**
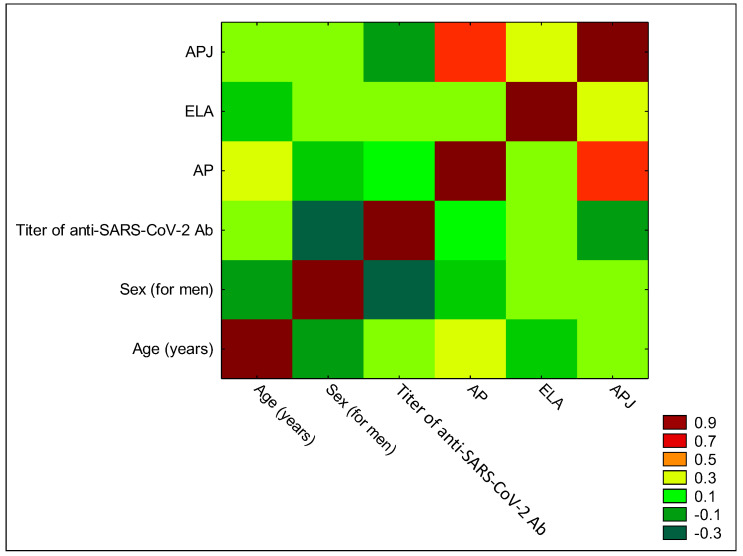
Heatmap for correlation coefficients of selected variables. Red color indicates a statistically significant correlation between AP and APJ (R = 0.566, *p* = 0.001).

We also analyzed whether there were any correlations between measured concentrations of AP, ELA, and APJ and age, sex, or titer of anti-SARS-CoV-2 antibodies. Our data, presented in Table 3 and Figure 5, showed a significant positive correlation between AP and APJ concentration (R = 0.566, *p* < 0.001).

## 4. Discussion

This paper describes changes in apelinergic system compounds, such as AP, ELA, and APJ, in plasma obtained from repeat blood donors at different time points since a SARS-CoV-2 infection, taking into account long-term changes after 120 days. The apelinergic system plays a significant role in maintaining cardiovascular function, fluid homeostasis, and metabolic regulation. It is also important to note that the apelinergic system compounds act, usually in a counterbalancing manner, with the renin–angiotensin–aldosterone system (RAAS). These well-documented interactions concern the regulation of blood pressure, vascular tone, and the progression of metabolic diseases such as hypertension. AP, a peptide initially isolated from bovine stomach extracts, has been shown to have widespread effects on various organ systems, including the cardiovascular, endocrine, and gastrointestinal systems. The role of AP in the cardiovascular system is of particular interest due to its ability to lower blood pressure and counteract the effects of Ang II [23]. The impact of COVID-19 on these systems has become a growing area of interest due to the fact that the SARS-CoV-2 virus binds to ACE2 to enter human cells. ACE2 is a crucial and critical enzyme to the RAAS that metabolizes Ang II into Ang 1–7, a vasodilatory molecule. Therefore, the downregulation of ACE2 during SARS-CoV-2 infection could disrupt the balance between the RAAS and the apelinergic system and may contribute to the cardiovascular complications seen in COVID-19 patients.

The present study was conducted in COVID-19 convalescents, in a group of repeat blood donors, which allowed us to eliminate the potential impact of chronic and acute cardiovascular diseases on the obtained results. Observation of the concentration of the apelinergic system components in the long term after the elimination of SARS-CoV-2 infection, in subsequent approximate 30-day intervals, up to 120 days from confirmation of infection and recovery, allowed us to observe changes that might be affecting the pathophysiology of diseases that are a late complication of SARS-CoV-2 infection.

On the other hand, considering the selection of patients for the study group, one of the study limitations is the fact that our study group was not homogeneous in terms of the concentration of anti-SARS-CoV-2 antibodies. Therefore, it should be further investigated whether the level of anti-SARS-CoV-2 antibodies affects the parameters of the apelinergic system.

Our study showed that AP levels remained lower in healthy convalescents after SARS-CoV-2 infection, both in the early post-infection period and 120 days after infection, compared to those who were never infected by SARS-CoV-2. An analogous trend was observed for ELA and APJ. There is also a significant positive correlation between AP and APJ levels (R = 0.566, *p* = 0001). In the early post-recovery period, both parameters decreased compared to noninfected people (*p* < 0.005). After a longer timeframe, 120 days after a positive SARS-CoV-2 PCR test, AP returned to a level not significantly different from that for uninfected individuals. Interestingly, APJ levels remained significantly lower, indicating a prolonged disruption of the apelinergic system. The persistent downregulation of APJ could have long-term implications for cardiovascular health, as this receptor is essential for mediating the effects of both AP and ELA.

Studies have shown that AP levels are lower in COVID-19 patients, especially those with hypertension and obesity [4,12,24,25,26], suggesting that apelinergic dysregulation could contribute to the severity of these patients’ symptoms. Rostamzadeh et al. [16] found that COVID-19 patients had significantly lower AP levels upon hospital admission, particularly if they were hypertensive or obese. This suggests that AP could be involved in the complex interactions between SARS-CoV-2 infection and pre-existing metabolic conditions. Berber et al. [27] also concluded that in COVID-19 patients, AP levels were lower compared to control groups, which is consistent with our findings. Lower serum AP levels in SARS-CoV-2-infected patients are likely a result of its degradation by ACE2, as circulating ACE2 has been shown to be increased in COVID-19 patients [28]. However, this pattern is not universal. Kenoosh et al. [15] observed increased AP levels in COVID-19 patients, attributing this to the reduced availability of ACE2 for Ang II metabolism. With more Ang II available, AP secretion is upregulated as a compensatory mechanism [9,15]. In a study by McGrail et al. [29], AP levels were increased in COVID-19 patients; however, they focused on the influence of cannabinoids in anti-COVID-19 therapy. This contrasting evidence highlights the need for further research to clarify the exact relationship between COVID-19 and the apelinergic system. The use of AP in the treatment of COVID-19 patients is also being considered, especially for patients with diseases predisposing them to a severe course of infection, such as diabetes, obesity, and hypertension. Due to the decrease in ACE2 concentration in SARS-CoV-2-infected patients, and, thus, the activation of ACE and Ang-II signaling, it can be concluded that AP has the potential to counteract respiratory and cardiovascular complications by regulating the growth of ACE2, while simultaneously increasing the Ang (1–7)/Ang-II ratio and suppressing Ang-II signaling. However, the increase in ACE2 availability increases susceptibility to SARS-CoV-2 infection, which is why AP or its analogs are proposed for use during the period of low SARS-CoV-2 viremia [8,28,30].

ELA, a relatively newer member of the apelinergic system, has been less studied than AP, but emerging evidence suggests that it also plays a critical role in cardiovascular homeostasis. ELA was first identified as essential for embryonic heart development, and more recent studies have implicated it in the maintenance of normal blood pressure and vascular health [31]. Decreased levels of ELA have been associated with pulmonary arterial hypertension and right ventricular hypertrophy, indicating that it has protective cardiovascular functions. Our study revealed a significant reduction in ELA concentration shortly after SARS-CoV-2 infection, in comparison to both healthy individuals and long-term post-infection cases (120 days after infection; *p* = 0.039 and *p* < 0.001, respectively). Although we examined both healthy individuals who recovered after SARS-CoV-2 infection and those who were never infected, rather than currently infected patients, ELA concentration was found to be lower in COVID-19 patients than in healthy people [17]. This suggests that the apelinergic system, including ELA, experiences acute disruption during the early stages of recovery from COVID-19. However, a study by Mermutluoglu et al. [32] contradicts these findings, showing no significant difference in ELA levels between COVID-19 patients and controls. The reasons for these dissimilarities remain unclear, though they may be due to differences in study populations, measurement techniques, or variations in the severity of illness among the patients studied. A plausible explanation for the observed reduction in AP and ELA levels post-COVID-19 infection is the virus’ interaction with ACE2. By binding to ACE2, the SARS-CoV-2 virus reduces the availability of this enzyme for its normal physiological functions, including the degradation of Ang II. This can lead to an overactivation of the RAAS and reduced AP signaling, which may account for the lower AP and ELA levels observed in some studies. Moreover, inflammation caused by SARS-CoV-2 infection could further suppress the expression of APJ, intensifying the dysregulation of the apelinergic system. The prolonged downregulation of APJ, as observed in our study, suggests that the effects of COVID-19 on this system may persist well beyond the early phase of infection.

The apelinergic system, which includes AP, ELA, and APJ, plays a critical role in cardiovascular and metabolic regulation. Disruptions to this system, particularly in the context of SARS-CoV-2 infection, can have significant health implications. Further research is, therefore, needed to fully understand the long-term effects of COVID-19 on the apelinergic system, as well as the potential therapeutic implications for targeting this system in COVID-19 patients with pre-existing metabolic and cardiovascular conditions.

## 5. Conclusions

The results obtained in the described study suggest that infection with the SARS-CoV-2 causes changes in some parameters of the apelinergic system, both in the short time period that has passed since the disease onset and even up to 4 months after infection. In order to understand the biological role of the apelinergic system in preventing the negative effects of cardiovascular deregulation, it is advisable to perform additional studies to unify our knowledge of the effects of SARS-CoV-2 on biochemical parameters important to the cardiovascular system. At the same time, there is a need to further study the parameters of the apelinergic system and determine its dependency on various factors, including viral infection.

## Figures and Tables

**Table 1:** Characteristics of repeat blood donors in the study group (*n* = 30) and control group (*n* = 30).

	Control Group *n* = 30	Study Group *n* = 30	*p*-Value
Gender:			0.717
Man	25 (83.3%)	26 (86.7%)	
Woman	5 (16.7%)	4 (13.3%)	
Age in years;			0.226
Median [IQR]	37 [34–42]	44 [32–47]	
Titer of anti-SARS-CoV-2 antibodies:	-		-
Low (<500)		10 (33.3%)	
High (>500)		20 (66.6%)	

**Table 2:** The concentrations of AP, ELA, and APJ in the serum of healthy control individuals and in repeat blood donors in Periods 1 and 4 after SARS-CoV-2 infection. Data are presented as median [IQR].

	Control Group (*n* = 30)	Period 1	Period 4	*p*-Value (Control vs. Period 1; Mann–Whitney Test)	*p*-Value (Control vs. Period 4; Mann–Whitney Test)	*p*-Value (Period 1 vs. Period 4, Post Hoc Test)
AP (pg/mL)	75.50 [59.50–126.16]	45.37 [30.60–77.91]	63.82 [49.30–92.70]	0.005 *	0.191	0.004 *
ELA (pg/mL)	1398.12 [923.75–1943.75]	1071.50 [920.00–1182.00]	1255.50 [1150.00–1365.00]	0.039 *	0.445	<0.001 *
APJ (ng/mL)	13.95 [5.43–20.05]	2.01 [0.96–5.39]	1.82 [1.00–4.96]	<0.001 *	<0.001 *	0.100

*: Statistically significant.

**Table 3:** Correlations between age, sex, and titer of anti-SARS-CoV-2 antibodies, and AP, ELA, and APJ concentrations.

	Age (Years)	Sex	Titer of Anti-SARS-CoV-2 Antibodies	AP	ELA	APJ
Age (years)	1					
Sex	R = −0.164 *p* = 0.385	1				
Titer of anti-SARS-CoV-2 antibodies	R = 0.102 *p* = 0.590	R = −0.346 *p* = 0.060	1			
AP	R = 0.283 *p* = 0.128	R = −0.022 *p* = 0.905	R = 0.057 *p* = 0.764	1		
ELA	R = −0.012 *p* = 0.945	R = 0.181 *p* = 0.337	R = 0.187 *p* = 0.319	R = 0.129 *p* = 0.495	1	
APJ	R = 0.123 *p* = 0.515	R = 0.101 *p* = 0.591	R = −0.38 *p* = 0.464	R = 0.566 *p* = 0.001 *	R = 0.285 *p* = 0.126	1

*: Statistically significant.

## Data Availability

The data are available from the corresponding author and may be shared if necessary.
